# Fatty Acid Metabolism and Derived-Mediators Distinctive of PPAR-α Activation in Obese Subjects Post Bariatric Surgery

**DOI:** 10.3390/nu13124340

**Published:** 2021-12-01

**Authors:** Claudia Manca, Stefano Pintus, Elisabetta Murru, Giovanni Fantola, Michela Vincis, Barbara Batetta, Enrico Moroni, Gianfranca Carta, Sebastiano Banni

**Affiliations:** 1 Department of Biomedical Sciences, University of Cagliari, 09042 Monserrato, Italy; claumanca@unica.it (C.M.); m.elisabetta.murru@gmail.com (E.M.); bbatetta@unica.it (B.B.); banni@unica.it (S.B.); 2 Bariatric Surgery Department, ARNAS Brotzu, 09121 Cagliari, Italy; stepintuss@gmail.com (S.P.); giovannifantola@aob.it (G.F.); michi_vin@yahoo.it (M.V.); enrico.moroni@aob.it (E.M.)

**Keywords:** obesity, bariatric surgery, non-esterified fatty acid (NEFA), *N*-oleoylethanolamine (OEA), peroxisome proliferator-activated receptor (PPAR)-α

## Abstract

Bariatric surger (BS) is characterized by lipid metabolic changes as a response to the massive release of non-esterified fatty acids (NEFA) from adipose depots. The study aimed at evaluating changes in polyunsaturated fatty acids (PUFA) metabolism and biosynthesis of the lipid mediators *N*-acylethanolamines (NAE), as indices of nuclear peroxisome proliferator-activated receptor (PPAR)-α activation. The observational study was performed on 35 subjects (27 female, 8 male) with obesity, undergoing bariatric surgery. We assessed plasma FA and NAE profiles by LC-MS/MS, clinical parameters and anthropometric measures before and 1 and 6 months after bariatric surgery. One month after bariatric surgery, as body weight and clinical parameters improved significantly, we found higher plasma levels of *N*-oleoylethanolamine, arachidonic and a 22:6-n3/20:5-n3 ratio as evidence of PPAR-α activation. These changes corresponded to higher circulating levels of NEFA and a steep reduction of the fat mass. After 6 months 22:6-n3/20:5-n3 remained elevated and fat mass was further reduced. Our data suggest that the massive release of NEFA from adipose tissue at 1-Post, possibly by inducing PPAR-α, may enhance FA metabolism contributing to fat depot reduction and improved metabolic parameters in the early stage. However, PUFA metabolic changes favor n6 PUFA biosynthesis, requiring a nutritional strategy aimed at reducing the n6/n3 PUFA ratio.

## 1. Introduction

The growing morbidity of obesity and its associated metabolic diseases are challenged with different strategies from reduced calorie intake in combination with physical activity, to radical changes towards a healthy lifestyle. Since overweight reduction and improvement of associated metabolic dysfunctions are very modest and few data support their long term maintenance [1], pharmacologic treatments are recommended when these approaches fail; however, many anti-obesity drugs have been withdrawn from the market due to their limited weight loss potential and serious adverse effects, such as cardiovascular events [2], gastrointestinal, hepatic and kidney injuries [3,4], mood disorders, and even suicidal susceptibility [5]. 

Bariatric surgery, causing food restriction and intestinal malabsorption, represents the most effective approach so far for the treatment of obesity, because it sustains weight loss and before that, often within days, improves glycemia [6] and other metabolic parameters e.g., triglycerides (TG) and HDL-cholesterol [7]. The most performed bariatric surgery includes Sleeve Gastrectomy (SG) and Roux-en-Y Gastric Bypass (RYGB). Both procedures, but mostly RYGB, can induce nutritional deficiencies [7].

One week after RYGB, enhanced insulin sensitivity and fasting hepatic insulin clearance was shown in 10 subjects with obesity and with or without type 2 diabetes [8]. In the same study, increased glucagon-like peptide 1 secretion and insulin sensitivity in the muscle and adipose tissue were observed from the third month to 1 year after RYGB [8]. 

The bidirectional communication between the gut and the brain, referred to as the ‘gut-brain axis’, links the emotional and cognitive centers of the brain with peripheral intestinal functions. This network includes the central nervous system, the autonomic nervous system, the enteric nervous system and the hypothalamic pituitary adrenal axis [9]. To maintain energy homeostasis the hypothalamus controls the gut-brain axis through several signaling molecules such as growth hormone, leptin, ghrelin, and the endocannabinoids (EC) *N*-arachidonoylethanolamine (AEA) and 2-arachidonoylglycerol (2-AG) [10,11,12]. Obesity, inflammation, insulin resistance and dyslipidemia are often associated to EC system hyper-tone, dysregulation of AEA congeners, and *N*-acylethanolamine (NAE) [13]. These molecules are considered controllers of energy balance and different metabolic parameters, peculiarly AEA, stimulates energy intake and storage [14], while the NAE *N*-oleoylethanolamine (OEA) mediates anorectic signals [15]. Changes in their levels have been observed in mice fed high fat high sugar diets for 56 days which showed glucose intolerance, since the day three, followed by weight gain and hyperinsulinemia [16]. 

In a human study including 65 patients with obesity that underwent laparoscopic SG surgery, Azar et al. followed the patients for up to 12 months and found postoperatively reduced levels of 2-AG, AEA, and arachidonic acid (20:4-n6); interestingly the delta of EC levels between pre- and post-operation were correlated with the delta of different metabolic parameters, waist circumference (WC), free fat mass (FFM), total cholesterol, and TG [17]. 

Insulin resistance related to obesity and associated pathologies is considered partly responsible for changes in circulating fatty acid (FA) levels [18], therefore is not surprising to observe alterations in EC and NAE levels, which depend on the availability of their respective diet-derived FA [19,20,21,22,23]. 

It is known that a hyperactive EC system is associated with obesity and relative metabolic disturbance, while little information is available whether the early ameliorations of metabolic parameters, which precede body weight and fat mass (FM) reduction, observed after bariatric surgery (as RYGB or SG), are related to changes EC and congeners. Therefore, in this study we aimed to evaluate the anthropometric data, metabolic parameters, circulating EC, NAE and FA prior to surgery and in early postoperative stages, at 1 and 6 months after bariatric surgery, in order to understand whether the massive release of NEFA from adipose tissue, by inducing PPAR-α, may enhance FA metabolism contributing to fat depot reduction and improved metabolic parameters in the early stage, and thereby shed light on the mechanisms underlying these ameliorations. 

## 2. Materials and Methods

### 2.1. Reagents

The acetonitrile, methanol, chloroform, n-hexane, ethanol, acetic acid, FA standards, all HPLC/MS grade, and deferoxamine mesylate were purchased from Sigma Chemicals Co. (St. Louis, MO, USA). Ascorbic acid, potassium hydroxide, and hydrochloric acid were purchased from Carlo Erba (Milano, Italy). FA standards including linoleic acid (18:2-n6), 20:4-n6, docosatetraenoic acid (22:4-n6), α-linolenic acid (18:3-n3), eicosapentaenoic acid (20:5-n3), docosahexaenoic acid (22:6-n3), palmitic acid (16:0), palmitoleic acid (16:1), stearic acid (18:0), and oleic acid (18:1-n9) were obtained from Sigma (Milan, Italy). Internal deuterated standards for the AEA, 2-AG, and OEA quantification by isotope dilution ([2H]^8^ AEA, [2H]^5^ 2-AG, [2H]^2^ OEA, *N*-palmitoylethanolamine [2H]^4^ PEA) were purchased from Cayman Chemicals (Ann Arbor, MI, USA).

### 2.2. Study Design

Changes in polyunsaturated fatty acids (PUFA) metabolism and lipid mediators NAE, as indices of nuclear peroxisome proliferator-activated receptor (PPAR)-α activation were evaluated in individuals with obesity scheduled to undergo bariatric surgery in order to understand mechanisms underlying body composition and, eventually, glucose and lipid metabolism ameliorations, up to 6 months after the surgery. 

### 2.3. Participants

Sixty-eight white Caucasian participants scheduled to undergo bariatric surgery were recruited at the Bariatric Surgery Centre, G. Brotzu Hospital (Cagliari, Italy) from February 2019 to September 2020. A subset of patients was enrolled in the study based on medical records and in-person interviews with a multidisciplinary team comprising surgical, nutritional, and psychological expertise. Diet counselling after bariatric surgery followed the clinical practice guidelines by the American Association of Clinical Endocrinologists, The Obesity Society, and American Society for Metabolic & Bariatric Surgery [24]. Patients diagnosed with major diseases (e.g., diabetes or kidney disease), food allergies, pregnant or breastfeeding, taking medications that could affect lipid metabolism were excluded from the study. 

**Scheme 1:**
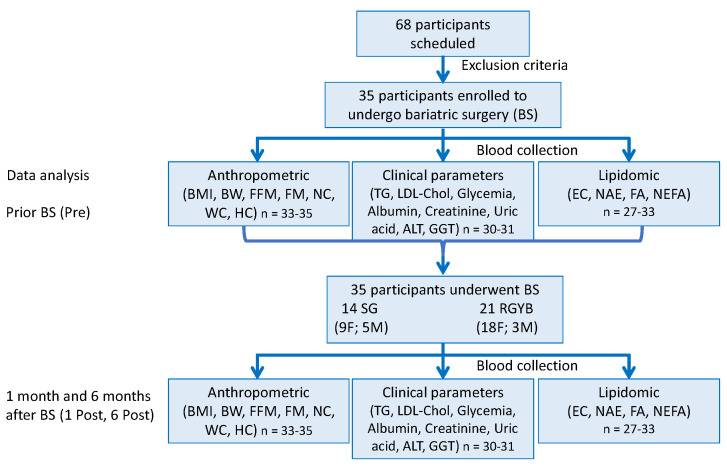
Study design and experimental procedure. Discrepancy in number of subjects, *n*, in data analysis are due to the limited sample amount available and/or their loss during the analysis procedure. Body mass index (BMI), free fat mass (FFM), fat mass (FM), neck (NC), waist (WC), hip (HC) circumferences, triglycerides (TG), LDL-cholesterol (LDL-Chol), alanine aminotransferase (ALT), gamma-glutamyl transferase (GGT), endocannabinoids (EC), N-acylethanolamine (NAE) non-esterified fatty acids (NEFA), fatty acids (FA).

Based on these criteria, thirty-five subjects (8 men and 27 women) with an average age of 41.9 y, body mass index (BMI) of 43.4 kg/m^2^ (range 33.1–58.5 kg/m^2^) participated in this study (Table 1); they were enrolled to undergo either SG (*n* = 14, 5 men and 9 women) or RYGB (*n* = 21, 3 men and 18 women). All bariatric surgery was performed by the same surgical team. The sample size was calculated a priori using a 0.01 significance level and 80% power, based on the assumption that the expected within-patient standard deviation was 0.05 and the minimal detectable difference in treatment means was of 0.03 units (20% difference in values) (G*Power 3.1.9.2). An overview of the study design is represented in Scheme 1. 

The study was conducted according to the guidelines of the Declaration of Helsinki and approved by the Ethics Committee of the University Hospital of Cagliari (Prot. PG/2019/1546 31.01.2019). Informed consent was obtained from all subjects involved in the study. This observational study was registered on ClinicalTrials.gov (accessed on 30 January 30) with the Identifier: NCT04984785.

### 2.4. Experimental Procedure

Each participant was tested in three separate visits: before (Pre), one month (1 Post) and six months (6 Post) after bariatric surgery. The same anthropometric measurements and experimental procedures were completed in all three visits. Specifically, neck, waist and hip circumferences were measured, and body weight and height were taken to calculate the BMI (kg/m^2^). Bioelectrical impedance analysis (BIA) was performed by Bodygram 101 Plus (Akern, Pontassieve, Italy); the instrument allowed measurements on subjects with weights of up to 200 kg. A constant current source at a frequency of 50 kHz and 8 mÅ was applied to estimate body composition.

Furthermore, in each visit, a sample of venous whole blood following overnight fasting, was collected and promptly centrifuged at 2000× *g* for 15 min at room temperature. From whole blood, haemoglobin and blood cell count were measured. While in plasma we determined LDL-Chol, TG, fasting glycemia, uric acid, creatinine, albumin, and alanine aminotransferase (ALT) and gamma-glutamyl transferase (GGT). Aliquots of plasma were stored at −80 °C for lipid analyses. 

### 2.5. Lipid Analyses

#### 2.5.1. Endocannabinoids and *N*-acylethanolamines

The total lipids were extracted from the plasma by a chloroform/methanol 2:1, (*v*/*v*) solution, and deuterated EC and NAE were added as internal standards for their quantification. Analyses of EC and NAE from chloroform were carried out by an Agilent 1100 HPLC system (Agilent, Palo Alto, CA, USA) equipped with a mass spectrometry Agilent Technologies QQQ triple quadrupole 6420 (LC-MS/MS) with an ESI source, using positive mode (ESI+) for the compounds and their deuterated homologs quantification as described in [25]. 

#### 2.5.2. Fatty Acid Analyses 

The total lipids were extracted by the method of Folch. Briefly, samples of human plasma (1 mL) were dissolved into a 2:1 chloroform-methanol solution containing 2 μg of vitamin E [26]. In order to obtain free FA, an aliquot of chloroform containing the lipid fraction of each sample was mildly saponified and analyzed by an Agilent 1100 HPLC system with a diode array detector (Palo Alto, CA, USA) as previously described in [27]. Since saturated FAs are transparent to UV detection, they were measured, after methylation, by an Agilent 6890 gas chromatograph (Palo Alto, CA, USA) as described in [28].

The n3 highly-unsaturated fatty acid (n3 HUFA) score was calculated as the percentage of the sum of n3 FA with 20 or more carbon atoms and three or more double bonds, divided by the sum of total FA with 20 or more carbon atoms and more than three double bonds [29]:

n3 HUFA score = (20:5-n3 + 22:6-n3 + 22:5-n3)/(20:5-n3 + 22:6-n3 + 22:5-n3 + 20:3-n6 + 20:4-n6 + 22:4-n6 + 22:5-n6 + 20:3-n9) × 100.

The ratio 16:1/18:2 was used as a marker of de novo lipogenesis because 16:1 has been shown to be produced by de novo lipogenesis [30] and is scarcely present in the diet, while 18:2, being an essential FA, may exclusively derive from the diet. In addition, since circulating non-esterified FA (NEFA) are mainly derived from adipose tissue in fasting humans, the ratio of 16:1/18:2 in NEFA could be considered an index of adipose de novo lipogenesis.

The ratio 22:6-n3/20:5-n3 was used as a marker of peroxisomal β-oxidation induced by PPAR-α, since 22:6-n3 biosynthesis requires a limiting step that is the β-oxidation in peroxisomes, which is finely regulated by the transcription factor PPAR-α [31,32]; an increase of the 22:6-n3/20:5-n3 ratio was taken as an indicator of PPAR-α activation [33,34]. 

### 2.6. Statistical Analysis 

Data are expressed as the mean ± SEM, specifically NEFA as nmoles/mL plasma, FA, EC and NAE as mol% of total FA. Unfortunately, we were not able to measure some plasma parameters in all subjects due to the limited sample amounts available and/or their loss during the analysis procedure. Because FA, EC, NAE clinical and anthropometric data were not normally distributed (Shapiro-Wilk normality test), the statistical significances among groups were assessed using the nonparametric Friedman One-Way Repeated Measures test followed by Dunn’s correction for multiple comparisons. Data evaluated for both surgical procedures, and stratified for gender, did not show relevant differences (data not shown), therefore data were analyzed as one group.

Data were analyzed using the GraphPad Prism 6.01 Software (La Jolla, CA, USA) with *p* ≤ 0.05 as the cut-off for statistical significance among groups.

## 3. Results

### 3.1. Body Composition and Blood Parameters

**Figure 1:**
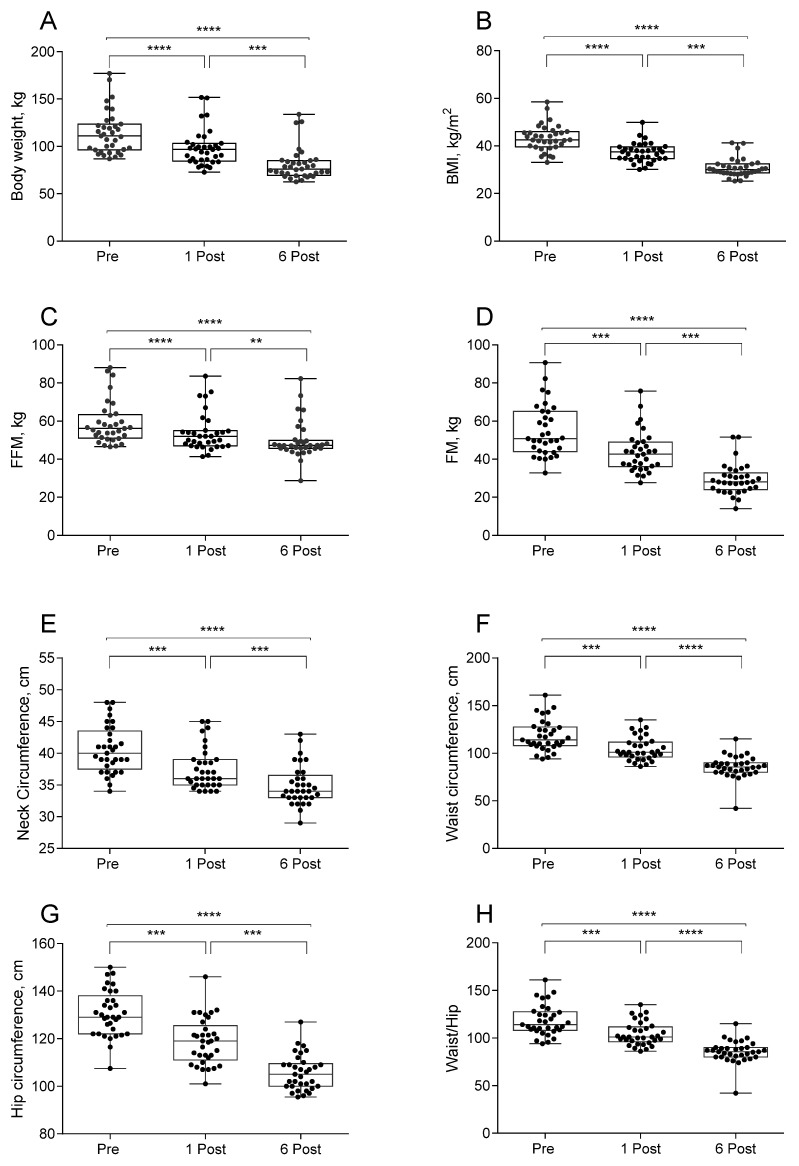
Values of body weight (**A**), body mass index (BMI, (**B**)), free fat mass (FFM, (**C**)), fat mass (FM, (**D**)), neck (**E**), waist (**F**), and hip (**G**) circumferences and waist-to-hip ratio (**H**) in subjects at baseline (Pre), one month (1 Post) and six months (6 Post) after bariatric surgery. Values are presented as boxes (mean value) and whiskers (higher and lower values) (Body weight *n* = 35; BMI *n* = 35; FFM kg *n* = 33; FM kg *n* = 33; NC *n* = 33; WC *n* = 33; HC *n* = 33). ** *p* < 0.01; *** *p* < 0.001; **** *p* < 0.0001.

Bariatric surgery strongly impacted on body weight and body composition. As reported in Figure 1, body weight, FFM and FM expressed as kg were strongly reduced at 1 month post-surgery (*n* = 33–35). BMI was reduced from 43.4, to 37.2 and 30.7 at 1 and 6 months after surgery, respectively. Six months after surgery, both FFM and FM decreased by 10 and 25 kg, respectively. Stratification for gender and surgical procedures did not show relevant differences (data not shown) as already previously shown [7]. Analysis of these parameters represented as a percentage of total body weight showed a decrease of FM and an increase in FFM (data not shown). Hip, waist, neck circumferences and waist-to-hip ratio followed the same pattern of body composition measurements and were reduced after surgery (Figure 1).

Plasma lipid profile improved in individuals who underwent bariatric surgery, as demonstrated by the reduced (23.5%) TG at 6 Post as well as LDL-Chol (15.9%) (Table 2) (*n* = 31). Fasting glycemia was reduced starting from the first month after surgery, returning within the physiological range, less than 100 mg/dL.

Bariatric surgery caused reduction of albumin from 1 Post, and not significant changes were observed between 1 and 6 Post, while uricemia, ALT and GGT were strongly reduced at 6 Post, creatine did not change (Table 2). 

### 3.2. Plasma Fatty Acid and Their Bioactive Derivatives

**Figure 2:**
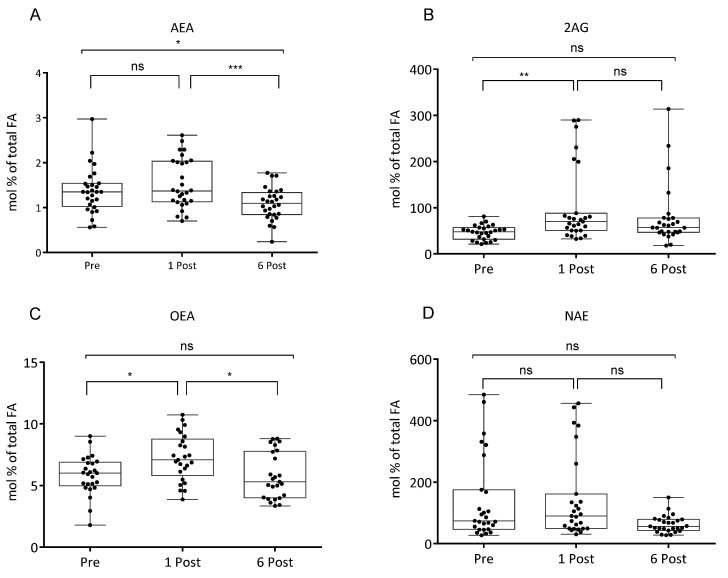
Values of *N*-arachidonoylethanolamine (AEA, (**A**)), 2-arachidonoylglycerol (2-AG, (**B**)), *N*-oleoylethanolamine (OEA, (**C**)), N-acylethanolamine (NAE, (**D**)), expressed as mol % of total fatty acid (FA), in subjects at baseline (Pre), one month (1 Post) and six months (6 Post) after bariatric surgery. Values are presented as boxes (mean value) and whiskers (higher and lower values) (AEA *n* = 28; 2AG *n* = 27; OEA *n* = 27; NAE *n* = 27). * *p* < 0.05; ** *p* < 0.01; *** *p* < 0.001.

EC (2-AG and AEA) and congeners (OEA, and PEA) plasma levels were modified by bariatric surgery as shown by LC-MS/MS analysis (Figure 2) (*n* = 25–28). OEA levels were increased after 1 month and returned to baseline at 6 Post. AEA levels were reduced at 6 Post, and total NAE had a similar but not significant pattern. Plasma levels of 2-AG were instead increased at 1 Post (Figure 2). 

**Figure 3:**
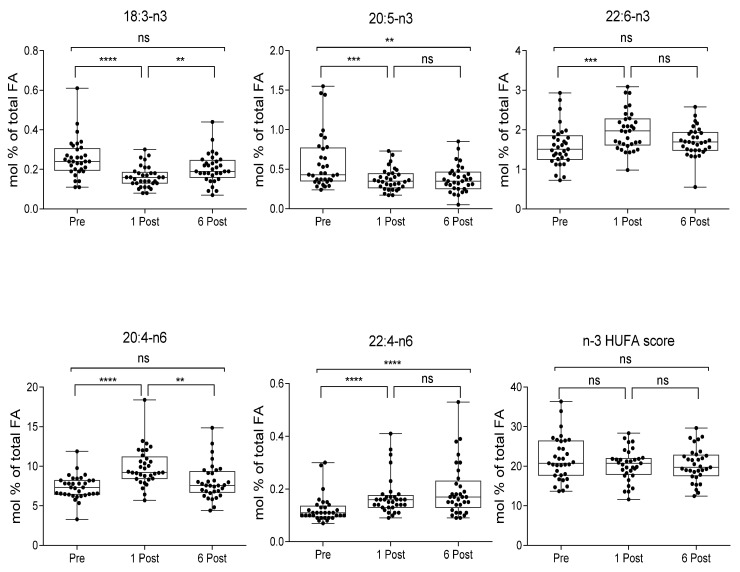
Plasma FA and *n3 HUFA scores in subjects at baseline (Pre), one month (1 Post) and six months (6 Post) after bariatric surgery. Values, expressed as mol % of total fatty acid (FA), are presented as boxes (mean value) and whiskers (higher and lower values) (*n* = 33). ** *p* < 0.01; *** *p* < 0.001; **** *p* < 0.0001.

Levels of long chain polyunsaturated FA appeared differently affected by bariatric surgery; 20:4-n6 and 22:6-n3, the major highly polyunsaturated FA n-6 and n-3, respectively, were enhanced at 1 Post and returned to basal levels at 6 Post (Figure 3) (*n* = 33). 

n3 HUFA score is calculated as the percentage of n3 HUFA (HUFA ≥ 20 carbons and ≥3 double bonds) in the total HUFA pool = (20:5-n3 + 22:6-n3 + 22:5-n3)/(20:5-n3 + 22:6-n3 + 22:5-n3 + 20:3-n6 + 20:4-n6 + 22:4-n6 + 22:5-n6 + 20:3-n9) × 100 [29].

**Figure 4:**
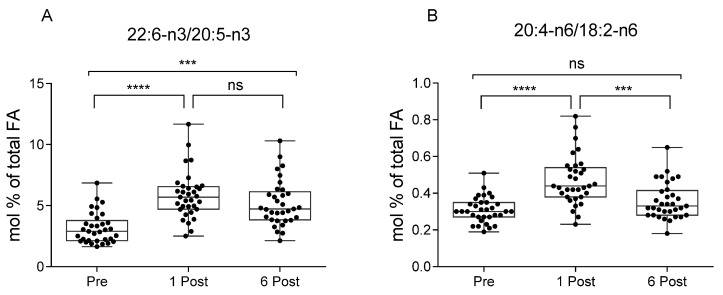
Ratios of 22:6-n3/20:5-n3 (**A**) [31,33], and 20:4-n6/18:2-n6 (**B**) [36] in subjects at baseline (Pre), one month (1 Post) and six months (6 Post) after bariatric surgery. Values, expressed as mol % of total fatty acid (FA), are presented as boxes (mean value) and whiskers (higher and lower values) (*n* = 33). *** *p* < 0.001; **** *p* < 0.0001.

The 22:6-n3/20:5-n3 ratio (a marker of PPAR-α activation [31,35]) and the 20:4-n6/18:2-n6 ratio, which represents an index of 18:2-n6 metabolism [36], were increased starting from 1 Post, an evidence of the enhanced n6 and n3 PUFA metabolism, and 22:6-n3/20:5-n3 ratio remained elevated at 6 Post (Figure 4) (*n* = 33). This was also confirmed by a steep reduction of 20:5-n3 and 18:3-n3, with the latter returning to baseline at 6 Post, and a progressive increase of 22:4 (Figure 3). n3 HUFA score levels remained relatively constant over the period considered.

**Figure 5:**
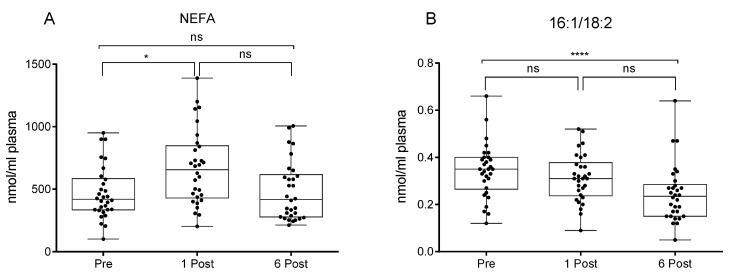
Values of non-esterified fatty acid (NEFA) (**A**) and 16:1/18:2 (**B**) [30] expressed as nmol/mL of plasma in participants at baseline (Pre), one month (1 Post) and six months (6 Post) after bariatric surgery. Values are presented as boxes (mean value) and whiskers (higher and lower values) (*n* = 30). * *p* < 0.05; **** *p* < 0.0001.

These changes occurred in parallel with higher circulating levels of NEFA (plus 56.6%) at 1 Post which returned to basal levels at 6 Post (Figure 5) (*n* = 33). The ratio of the NEFAs 16:1/18:2, considered an index of de novo lipogenesis, was instead strongly reduced at 6 Post with respect to the baseline. 

## 4. Discussion

In line with data in the literature we observed a strong body weight reduction from the first month; specifically, the FM reduction (15.8% *p* < 0.0001) was combined with a less severe reduction of FFM (8.2% *p* < 0.0001). Waist and neck circumferences are recognized as useful indicators of visceral fat accumulation [37]; accordingly, both neck circumference and FM were significantly decreased starting from 1 Post. 

It has been well established that bariatric surgery improves metabolism [6,7,38]. In the present study, TG and LDL-Chol profile reduction was observed at 6 Post after body weight loss, while from 1 Post, we observed a decrease in the glycemia concurrently to body weight loss, suggesting that may be independent from fat mass reduction. 

Reduced food intake and protein malabsorption may explain the significant decrease of albumin observed at 1 Post (3.88 g/dL plasma) and at 6 Post (3.95 g/dL plasma), however, values remained within the physiological range (3.4–5.4 g/dL) throughout the observation period, suggesting a non-relevant protein deficiency in the time frame taken into consideration. 

It is known there is a mutual association between obesity and hyperuricemia [39]. Hyperuricemia may derive from a decreased renal excretion due to chronic renal failure linked to increased BMI [40] and/or an excess of fructose intake in insulin-resistance patients [41]. In addition, uric acid may promote fat storage [42]. In our study, uricemia values were significantly reduced at 6 Post (4.47 mg/dL plasma) to physiological values (4–8 mg/dL), that might suggest rather than an amelioration of renal function, a metabolic improvement and/or a reduction of fructose intake [43,44]. 

Serum GGT and ALT are common hepatic inflammatory markers altered in obesity, non-alcoholic fatty liver disease (NAFLD) and fatty liver [45]. Their reduction at 6 Post to physiological values may suggest a significant improvement of obese-related liver dysfunction. 

Our data show that, in agreement with previous observation [18], alterations in FA metabolism in response to obesity surgery may be associated to several factors such as dietary changes and a modified enzymatic activity, mainly due to a different insulin response. High plasma levels of long chain PUFA such as 20:4-n6, 22:4-n6 and 22:6-n3, the simultaneous reduction of 18:3-n3 and 20:5-n3, precursors of 22:6-n3, and the elevated ratio of 20:4-n6/18:2-n6 suggest an enhanced n6 and n3 PUFA metabolism. Similarly, we observed an elevated 22:6-n3/20:5-n3 ratio, considered a marker of increased peroxisomal β-oxidation [31], a limiting step induced by activation of PPAR-α, which has important roles in the regulation of nutrient metabolism, including FA oxidation, gluconeogenesis, and amino acid metabolism [46]. Likely, PPAR-α enhanced activity might be ascribed to a 56.6% increase of circulating NEFA observed at 1 Post, likely due to its massive release from adipose tissue caused by dietary restriction which, by inducing PPAR-α, may enhance FA oxidation and contribute to fat depot reduction and improved metabolic and inflammatory parameters. In addition, sustained induction of PPAR-α may also reduce inflammation by catabolizing pro-inflammatory eicosanoids [31].

NEFA levels, mainly from visceral adipose tissue [47], are usually steadily elevated in the plasma of subjects with obesity irrespective of fasting conditions, due to the insulin resistance. However, data in the literature conflict with some studies describing a decrease in plasma NEFA levels [48], and others describing no changes [49]. The increase found in circulating NEFA at 1 Post, which returned to pre surgery values at 6 Post, may suggest that at 1 Post NEFA release from adipose tissue prevails the capacity of liver and other tissues uptake, or alternatively there is a massive release due to the severe calorie restriction in the immediate post-surgery. Interestingly, analysis of the NEFA profile showed that 16:1/18:2 ratio was reduced at 6 Post. Despite 16:0 and 16:0/18:2 having been identified as the biomarkers of de novo lipogenesis [50], 16:1 has also been shown to be a marker of de novo lipogenesis [30] and most likely released as NEFA from adipose tissue. Therefore, the 16:1/18:2 ratio in NEFA could also be considered an index of adipose de novo lipogenesis, suggesting that reduced glycemia may result in less substrate availability for de novo lipogenesis, further favoring fat mass reduction. 

**Figure 6:**
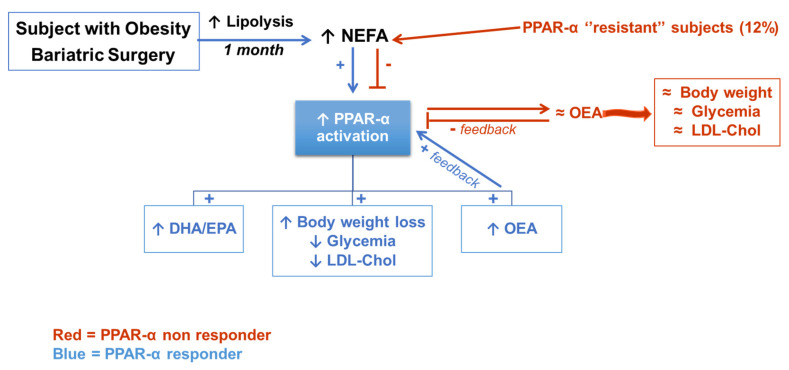
The increased NEFA released from adipose tissue may activate PPAR-α which induces PUFA metabolism, FA β-oxidation and increased circulating OEA levels further enhance PPAR-α activation and hence induce body weight loss and improved glucose and lipid metabolism. In red the putative mechanism which may explain why four subjects (12%) did not respond in the same way to circulating NEFA, leading us to hypothesize a ’’resistance’’ to PPAR-α activation. Red, PPAR-α non responder subjects; Blue, PPAR-α responder subjects; ↑, increase; ↓, decrease; +, positive regulation; -, negative regulation; ≈, unchanged.

Activation of PPAR-α by increased NEFA levels at 1 Post may induce a plethora of metabolic changes such as the activation of enzymes involved in FA metabolism with a consequent increased 20:4-n6 and 22:6-n3 biosynthesis [51], and a raise of the NAE OEA levels [52], which may further induce PPAR-α and thereby mitigate hunger and promote FA β-oxidation. Indeed, we previously demonstrated in experimental models that PPAR-α activation resulted in OEA increase, which further sustained PPAR-α activation by a positive feedback mechanism [52]. As a matter of fact, OEA was shown to reduce the accumulation of visceral fat by activation of PPAR-α [19,20,21,22,23]. The reduction of OEA at 6 Post may be ascribed to the minor induction of PPAR-α by the lower NEFA release from adipose tissue. It would be interesting to envisage a strategy to induce PPAR-α at 6 Post by nutritional [53] or pharmacological [54] treatments to evaluate whether OEA and 22:6-n3 levels will return to the high values found at 1 Post and further positively influence lipid and energy metabolism and perhaps reduce relapse incidence (Figure 6). 

Remarkably, only in 4 patients were OEA levels at 1 Post not increased (non responders –0.92 ± 1.38 responders 0.84 ± 2.92 Mean ± SD of difference from Pre at 1 month) despite the higher levels of NEFA and they revealed lesser than 7% of fat mass reduction at 6 Post (non responders –5.14 ± 2.90 Responders –11.97 ± 3.17 Mean + SD of difference from Pre at 6 month), mainly visceral as indicated by corresponding values of waist circumference, suggesting that these subjects may be regarded as non PPAR-α responders, probably characterized by some sort of a “PPAR-α resistance”. The geographically and ethnically homogenous cohort may represent a limitation of this study; it would be therefore crucial to evaluate in a larger multicenter study whether OEA and NEFA levels may predict the incidence of relapse, where OEA levels at 1 month post-surgery may represent an early biomarker of relapse susceptibility, requiring personalized treatments to overcome PPAR-α lower responsiveness. However, NEFA binding to PPAR families other than PPAR-α may not be ruled out [55], therefore further studies should be devoted to investigating possible activation of other PPAR isoforms.

Substantial anatomical changes along with reduced caloric intake and absorption imposed by bariatric surgery lead to a new metabolic balance to sustain fat mass loss. Our findings propose the contribution of FA-derived mediators on metabolic amelioration, improved lipid profile, reduction of glycemia, and fat mass. Indeed, it has been observed that NAE and specifically OEA reduces fat mass in rodents, possibly by activating PPAR-α and, even if human studies are limited, OEA supplementation in humans was shown to promote weight loss therapy [20]. Nonparametric Spearman correlation analyses considering the percentage of 1 Post (which was the time with major changes) versus Pre bariatric surgery conditions (as 100%) identified a negative trend (r = –0.348 *p* = 0.059) between OEA and BMI and between OEA and body weight (r = –0.335 *p* = 0.07).

Interestingly, increase of 20:4-n6 at 1 Post paralleled the increased levels of 2-AG, yet the latter remained elevated at 6 Post despite reduced levels of its precursor, while AEA was found reduced. However, as we recently demonstrated in animal models, AEA plasma levels were strongly correlated to their precursor levels in adipose tissue, liver and brain but not in plasma [56]. 2-AG plasma levels were shown to be correlated with increased appetite, especially for calorie dense foods [57], therefore higher levels may not be desirable. Previously we showed that 2-AG plasma levels may be successfully reduced by krill oil as a source of 20:5-n3 and 22:6-n3 [58]. A return to Pre levels of OEA, several PUFA and NEFA at six months along with reduction of AEA and, possibly, lipogenesis at six months post, may suggest the need of a dietary intervention in order to improve the long term effect of bariatric surgery on the metabolic and FA profile and energy metabolism. Our data show a low n3 HUFA score, which is a potential blood biomarker of n3 FA intake and tissue status, in all the groups we considered; indeed values around 20 are considered relatively low typical of low fish consumption [29,59]. Thus, n3 PUFA supplementation may be envisaged to patients undergoing bariatric surgery and particularly to those with 2-AG higher levels and tendency to high consumption of calorie dense foods as a strategy to avoid or limit relapse of morbid obesity.

## 5. Conclusions

FA metabolism may contribute to early metabolic changes occurring after bariatric surgery, such as improved glycemia, lipid blood profile, reduced body weight and fat mass, by modulating lipid mediator biosynthesis, in particular increasing OEA levels, which activate specific receptors such as PPAR-α. Indeed, our data show that the massive release of NEFA from adipose tissue, possibly inducing PPAR-α, may enhance FA metabolism and contribute to fat depot reduction and improved metabolic parameters. However, PUFA metabolic changes favor n6 PUFA biosynthesis, requiring a nutritional strategy aimed at significantly reducing the ratio of n6/n3 PUFA to further ameliorate the FA profile and energy metabolism. Therefore, a personalized nutritional approach may be proposed to further sustain the response to bariatric surgery in order to reduce the relapse.

## Figures and Tables

**Table 1:** Main anthropometric characteristics of subjects at baseline before bariatric surgery.

	Female		Male	
Surgery (*n*)	SG (9)	RYGB (18)	SG (5)	RYGB (3)
Age (y)	41.0 ± 1.98		44.6 ± 3.60	
BMI (kg/m^2^)	42.7 ± 0.91		45.7 ± 2.63	
Weight (kg)	107.3 ± 2.96		138.4 ± 9.91	
FM (kg)	53.0 ± 2.18		60.6 ± 6.90	
FFM (kg)	54.3 ± 0.94		77.9 ± 3.17	

Values are means ± SEM. Number of subjects (*n*), age (y), Roux-en-Y Gastric Bypass (RYGB), Sleeve Gastrectomy (SG), body mass index (BMI), fat mass (FM), free fat mass (FFM).

**Table 2:** Plasma parameters in subjects at baseline (Pre), one month (1 Post) and six months (6 Post) after bariatric surgery.

	PRE		1 POST		6 POST	
	Mean	SEM	Mean	SEM	Mean	SEM
TRIGLYCERIDES, mg/dL	99.3	7.55 ^a^	89.6	5.34 ^a^	76.0	5.56 ^b^
LDL-CHOLESTEROL, mg/dL	128.6	6.24 ^a^	113.3	5.54 ^a^	108.2	6.22 ^b^
GLYCEMIA, mg/dL	107.3	4.67 ^a^	96.0	3.07 ^b^	88.4	2.27 ^b^
ALBUMIN, g/dL	4.2	0.26 ^a^	3.9	0.05 ^b^	3.9	0.07 ^b^
URICEMIA, mg/dL	5.9	0.22 ^a^	5.6	0.33 ^a^	4.5	0.29 ^b^
CREATININE, MG/DL	0.76	0.001 ^a^	0.79	0.01 ^a^	0.75	0.01 ^a^
ALT, IU/dL	30.2	3.17 ^a^	44.3	7.84 ^a^	18.9	1.84 ^b^
GGT, IU/dL	26.8	3.50 ^a^	25.1	2.36 ^a^	14.3	1.35 ^b^

Values are means ± SEM. Different letters indicate significant differences at *p* < 0.05 within each row, ^a^ represents a higher value with respect to ^b^. Triglycerides *n* = 31; LDL-cholesterol *n* = 30; Glycemia *n* = 31; Albumin *n* = 30; Uricemia *n* = 30; Creatinine *n* = 30; alanine aminotransferase (ALT) *n* = 30; gamma-glutamyl transferase (GGT) *n* = 30.

## Data Availability

The data presented in this study are available on request from the corresponding author. The data are not publicly available in accordance with consent provided by participants on the use of confidential data.

## Abbreviations

*N*-arachidonoylethanolamine (AEA); Alanine aminotransferase (ALT); Body weight (BMI); endocannabinoids (EC); Fat Mass (FM); fatty acids (FA); Free Fat Mass (FFM); gamma-glutamyl transferase (GGT), hip circumference (HC); LDL-cholesterol (LDL-Chol); *N*-acylethanolamine (NAE); neck circumference (NC); non-esterified fatty acids (NEFA); peroxisome proliferator-activated receptor-α (PPAR)-α; polyunsaturated fatty acids (PUFA); triglycerides (TG); waist circumference (WC)

## References

[1] Phelan S., Wing R.R. (2005). Prevalence of Successful Weight Loss. Arch. Intern. Med..

[2] Kang J.G., Park C.-Y. (2012). Anti-Obesity Drugs: A Review about Their Effects and Safety. Diabetes Metab. J..

[3] Yanovski S.Z., Yanovski J. (2014). Long-term drug treatment for obesity: A systematic and clinical review. JAMA.

[4] Filippatos T.D., Derdemezis C.S., Gazi I.F., Nakou E.S., Mikhailidis D.P., Elisaf M.S. (2008). Orlistat-associated adverse effects and drug interactions: A critical review. Drug Saf..

[5] Dietrich M.O., Horvath T.L. (2012). Limitations in anti-obesity drug development: The critical role of hunger-promoting neurons. Nat. Rev. Drug Discov..

[6] Isbell J.M., Tamboli R.A., Hansen E.N., Saliba J., Dunn J.P., Phillips S.E., Marks-Shulman P.A., Abumrad N.N. (2010). The Importance of Caloric Restriction in the Early Improvements in Insulin Sensitivity After Roux-en-Y Gastric Bypass Surgery. Diabetes Care.

[7] Kashyap S.R., Bhatt D.L., Wolski K., Watanabe R.M., Abdul-Ghani M., Abood B., Pothier C.E., Brethauer S., Nissen S., Gupta M. (2013). Metabolic Effects of Bariatric Surgery in Patients With Moderate Obesity and Type 2 Diabetes: Analysis of a randomized control trial comparing surgery with intensive medical treatment. Diabetes Care.

[8] Bojsen-Møller K.N., Dirksen C., Jørgensen N.B., Jacobsen S.H., Serup A.K., Albers P.H., Hansen D.L., Worm D., Naver L., Kristiansen V.B. (2013). Early Enhancements of Hepatic and Later of Peripheral Insulin Sensitivity Combined With Increased Postprandial Insulin Secretion Contribute to Improved Glycemic Control After Roux-en-Y Gastric Bypass. Diabetes.

[9] Carabotti M., Scirocco A., Maselli M.A., Severi C. (2015). The gut-brain axis: Interactions between enteric microbiota, central and enteric nervous systems. Ann. Gastroenterol..

[10] Briggs D.I., Enriori P.J., Lemus M.B., Cowley M., Andrews Z.B. (2010). Diet-Induced Obesity Causes Ghrelin Resistance in Arcuate NPY/AgRP Neurons. Endocrinology.

[11] Enriori P.J., Evans A.E., Sinnayah P., Jobst E.E., Tonelli-Lemos L., Billes S.K., Glavas M.M., Grayson B.E., Perello M., Nillni E.A. (2007). Diet-Induced Obesity Causes Severe but Reversible Leptin Resistance in Arcuate Melanocortin Neurons. Cell Metab..

[12] Guijarro A., Osei-Hyiaman D., Harvey-White J., Kunos G., Suzuki S., Nadtochiy S., Brookes P., Meguid M.M. (2008). Sustained Weight Loss After Roux-en-Y Gastric Bypass Is Characterized by Down Regulation of Endocannabinoids and Mitochondrial Function. Ann. Surg..

[13] Gatta-Cherifi B., Cota D. (2015). New insights on the role of the endocannabinoid system in the regulation of energy balance. Int. J. Obes..

[14] Di Marzo V., Matias I. (2005). Endocannabinoid control of food intake and energy balance. Nat. Neurosci..

[15] Fu J., Gaetani S., Oveisi F., Verme J.L., Serrano A., De Fonseca F.R., Rosengarth A., Luecke H., Di Giacomo B., Tarzia G. (2003). Oleylethanolamide regulates feeding and body weight through activation of the nuclear receptor PPAR-α. Nature.

[16] Lacroix S., Pechereau F., Leblanc N., Boubertakh B., Houde A., Martin C., Flamand N., Silvestri C., Raymond F., Di Marzo V. (2019). Rapid and Concomitant Gut Microbiota and Endocannabinoidome Response to Diet-Induced Obesity in Mice. mSystems.

[17] Azar S., Sherf-Dagan S., Nemirovski A., Webb M., Raziel A., Keidar A., Goitein D., Sakran N., Shibolet O., Tam J. (2018). Circulating Endocannabinoids Are Reduced Following Bariatric Surgery and Associated with Improved Metabolic Homeostasis in Humans. Obes. Surg..

[18] Walle P., Takkunen M., Männistö V., Vaittinen M., Käkelä P., Ågren J., Schwab U., Lindström J., Tuomilehto J., Uusitupa M. (2017). Alterations in fatty acid metabolism in response to obesity surgery combined with dietary counseling. Nutr. Diabetes.

[19] Bowen K.J., Kris-Etherton P.M., Shearer G.C., West S.G., Reddivari L., Jones P.J. (2017). Oleic acid-derived oleoylethanolamide: A nutritional science perspective. Prog. Lipid Res..

[20] Brown J.D., Azari E.K., Ayala J.E. (2017). Oleoylethanolamide: A fat ally in the fight against obesity. Physiol. Behav..

[21] Piomelli D. (2013). A fatty gut feeling. Trends Endocrinol. Metab..

[22] Sihag J., Jones P.J.H. (2017). Oleoylethanolamide: The role of a bioactive lipid amide in modulating eating behaviour. Obes. Rev..

[23] TuTunchi H., Ostadrahimi A., Saghafi-Asl M., Maleki V. (2019). The effects of oleoylethanolamide, an endogenous PPAR-α agonist, on risk factors for NAFLD: A systematic review. Obes. Rev..

[24] Mechanick J.I., Youdim A., Jones D.B., Garvey W.T., Hurley D.L., McMahon M.M., Heinberg L.J., Kushner R.F., Adams T.D., A Shikora S. (2013). Clinical practice guidelines for the perioperative nutritional, metabolic, and nonsurgical support of the bariatric surgery patient--2013 update: Cosponsored by American Association of Clinical Endocrinologists, The Obesity Society, and American Society for Metabolic & Bariatric Surgery. Obesity.

[25] Manca C., Carta G., Murru E., Abolghasemi A., Ansar H., Errigo A., Cani P.D., Banni S., Pes G.M. (2021). Circulating fatty acids and endocannabinoidome-related mediator profiles associated to human longevity. GeroScience.

[26] Folch J., Lees M., Sloane Stanley G.H. (1957). A simple method for the isolation and purification of total lipides from animal tissues. J. Biol. Chem..

[27] Banni S., Carta G., Contini M.S., Angioni E., Deiana M., Dessì M.A., Melis M.P., Corongiu F.P. (1996). Characterization of conjugated diene fatty acids in milk, dairy products, and lamb tissues. J. Nutr. Biochem..

[28] Batetta B., Griinari M., Carta G., Murru E., Ligresti A., Cordeddu L., Giordano E., Sanna F., Bisogno T., Uda S. (2009). Endocannabinoids May Mediate the Ability of (n-3) Fatty Acids to Reduce Ectopic Fat and Inflammatory Mediators in Obese Zucker Rats. J. Nutr..

[29] Stark K.D. (2007). The Percentage of n-3 Highly Unsaturated Fatty Acids in Total HUFA as a Biomarker for Omega-3 Fatty Acid Status in Tissues. Lipids.

[30] Volk B.M., Kunces L.J., Freidenreich D.J., Kupchak B.R., Saenz C., Artistizabal J.C., Fernandez M.L., Bruno R., Maresh C.M., Kraemer W.J. (2014). Effects of Step-Wise Increases in Dietary Carbohydrate on Circulating Saturated Fatty Acids and Palmitoleic Acid in Adults with Metabolic Syndrome. PLoS ONE.

[31] Ferdinandusse S., Denis S., Mooijer P.A., Zhang Z., Reddy J.K., Spector A.A., Wanders R.J. (2001). Identification of the peroxisomal β-oxidation enzymes involved in the biosynthesis of docosahexaenoic acid. J. Lipid Res..

[32] Tahri-Joutey M., Andreoletti P., Surapureddi S., Nasser B., Cherkaoui-Malki M., Latruffe N. (2021). Mechanisms Mediating the Regulation of Peroxisomal Fatty Acid Beta-Oxidation by PPARα. Int. J. Mol. Sci..

[33] Murru E., Carta G., Cordeddu L., Melis M.P., Desogus E., Ansar H., Chilliard Y., Ferlay A., Stanton C., Coakley M. (2018). Dietary Conjugated Linoleic Acid-Enriched Cheeses Influence the Levels of Circulating n-3 Highly Unsaturated Fatty Acids in Humans. Int. J. Mol. Sci..

[34] Saba F., Sirigu A., Pillai R., Caria P., Cordeddu L., Carta G., Murru M.E., Sogos V., Banni S. (2017). Downregulation of inflammatory markers by conjugated linoleic acid isomers in human cultured astrocytes. Nutr. Neurosci..

[35] Hsiao W.-T., Su H.-M., Su K.-P., Chen S.-H., Wu H.-P., You Y.-L., Fu R.-H., Chao P.-M. (2019). Deficiency or activation of peroxisome proliferator-activated receptor α reduces the tissue concentrations of endogenously synthesized docosahexaenoic acid in C57BL/6J mice. Nutr. Res. Pr..

[36] Martinelli N., Girelli D., Malerba G., Guarini P., Illig T., Trabetti E., Sandri M., Friso S., Pizzolo F., Schaeffer L. (2008). FADS genotypes and desaturase activity estimated by the ratio of arachidonic acid to linoleic acid are associated with inflammation and coronary artery disease. Am. J. Clin. Nutr..

[37] Borel A.-L., Coumes S., Reche F., Ruckly S., Pépin J.L., Tamisier R., Wion N., Arvieux C. (2018). Waist, neck circumferences, waist-to-hip ratio: Which is the best cardiometabolic risk marker in women with severe obesity? The SOON cohort. PLoS ONE.

[38] Schauer P.R., Bhatt D.L., Kirwan J.P., Wolski K., Aminian A., Brethauer S.A., Navaneethan S.D., Singh R.P., Pothier C.E., Nissen S.E. (2017). Bariatric Surgery versus Intensive Medical Therapy for Diabetes—5-Year Outcomes. N. Engl. J. Med..

[39] Chen Y., Zhang N., Sun G., Guo X., Yu S., Yang H., Zheng L., Sun Y. (2016). Metabolically healthy obesity also has risk for hyperuricemia among Chinese general population: A cross-sectional study. Obes. Res. Clin. Pr..

[40] Kalaitzidis R.G., Siamopoulos K.C. (2011). The role of obesity in kidney disease: Recent findings and potential mechanisms. Int. Urol. Nephrol..

[41] Kaneko C., Ogura J., Sasaki S., Okamoto K., Kobayashi M., Kuwayama K., Narumi K., Iseki K. (2017). Fructose suppresses uric acid excretion to the intestinal lumen as a result of the induction of oxidative stress by NADPH oxidase activation. Biochim. Biophys. Acta Gen. Subj..

[42] Choi Y.-J., Shin H.-S., Choi H.S., Park J.-W., Jo I., Oh E.-S., Lee K.-Y., Lee B.-H., Johnson R.J., Kang D.-H. (2014). Uric acid induces fat accumulation via generation of endoplasmic reticulum stress and SREBP-1c activation in hepatocytes. Lab. Investig..

[43] Nakagawa T., Hu H., Zharikov S., Tuttle K., Short R.A., Glushakova O., Ouyang X., Feig D., Block E.R., Herrera-Acosta J. (2006). A causal role for uric acid in fructose-induced metabolic syndrome. Am. J. Physiol. Physiol..

[44] McChesney M.J. (2016). Relationship between High-Fructose Corn Syrup, Uric Acid, and Metabolic Syndrome. J. Pediatr. Surg. Nurs..

[45] Johansson H.-E., Haenni A., Zethelius B. (2013). Platelet Counts and Liver Enzymes after Bariatric Surgery. J. Obes..

[46] Kersten S. (2002). Peroxisome proliferator activated receptors and obesity. Eur. J. Pharmacol..

[47] Björntorp P., Bergman H., Varnauskas E. (2009). Plasma free fatty acid turnover rate in obesity. Acta Med. Scand..

[48] Chearskul S., Delbridge E., Shulkes A., Proietto J., Kriketos A. (2008). Effect of weight loss and ketosis on postprandial cholecystokinin and free fatty acid concentrations. Am. J. Clin. Nutr..

[49] Keogh J.B., Luscombe-Marsh N.D., Noakes M., Wittert G.A., Clifton P.M. (2007). Long-term weight maintenance and cardiovascular risk factors are not different following weight loss on carbohydrate-restricted diets high in either monounsaturated fat or protein in obese hyperinsulinaemic men and women. Br. J. Nutr..

[50] Chong M.F.-F., Hodson L., Bickerton A.S., Roberts R., Neville M., Karpe F., Frayn K.N., A Fielding B. (2008). Parallel activation of de novo lipogenesis and stearoyl-CoA desaturase activity after 3 d of high-carbohydrate feeding. Am. J. Clin. Nutr..

[51] Rakhshandehroo M., Knoch B., Müller M., Kersten S. (2010). Peroxisome Proliferator-Activated Receptor Alpha Target Genes. PPAR Res..

[52] Melis M., Carta G., Pistis M., Banni S. (2013). Physiological Role of Peroxisome Proliferator-Activated Receptors Type Alpha on Dopamine Systems. CNS Neurol. Disord.-Drug Targets.

[53] Murru E., Carta G., Manca C., Sogos V., Pistis M., Melis M., Banni S. (2021). Conjugated Linoleic Acid and Brain Metabolism: A Possible Anti-Neuroinflammatory Role Mediated by PPARα Activation. Front. Pharmacol..

[54] Ferri N., Corsini A., Sirtori C., Ruscica M. (2017). PPAR-α agonists are still on the rise: An update on clinical and experimental findings. Expert Opin. Investig. Drugs.

[55] Pawar A., Jump D.B. (2003). Unsaturated Fatty Acid Regulation of Peroxisome Proliferator-activated Receptor α Activity in Rat Primary Hepatoctes. J. Biol. Chem..

[56] Murru E., Lopes P., Carta G., Manca C., Abolghasemi A., Guil-Guerrero J., Prates J., Banni S. (2021). Different Dietary N-3 Polyunsaturated Fatty Acid Formulations Distinctively Modify Tissue Fatty Acid and N-Acylethanolamine Profiles. Nutrients.

[57] Monteleone P., Piscitelli F., Scognamiglio P., Monteleone A.M., Canestrelli B., Di Marzo V., Maj M. (2012). Hedonic Eating Is Associated with Increased Peripheral Levels of Ghrelin and the Endocannabinoid 2-Arachidonoyl-Glycerol in Healthy Humans: A Pilot Study. J. Clin. Endocrinol. Metab..

[58] Banni S., Carta G., Murru E., Cordeddu L., Giordano E., Sirigu A.R., Berge K., Vik H., Maki K.C., Di Marzo V. (2011). Krill oil significantly decreases 2-arachidonoylglycerol plasma levels in obese subjects. Nutr. Metab..

[59] Bell J.G., Mackinlay E.E., Dick J.R., Younger I., Lands B., Gilhooly T. (2011). Using a fingertip whole blood sample for rapid fatty acid measurement: Method validation and correlation with erythrocyte polar lipid compositions in UK subjects. Br. J. Nutr..

